# Perioperative Outcomes of Acute Type-A Aortic Dissection Repair was Unaffected by COVID-19 Testing Delay

**DOI:** 10.26502/fccm.92920248

**Published:** 2022-04-05

**Authors:** Felix Orelaru, Elizabeth L Norton, Rana-Armaghan Ahmad, Aroma Naeem, Karen M Kim, Shinichi Fukuhara, Himanshu J Patel, G Michael Deeb, Bo Yang

**Affiliations:** 1 Department of General Surgery, St. Joseph Mercy, Ann Arbor, Michigan, USA; 2 Division of Cardiothoracic Surgery, Department of Surgery, Emory University, Atlanta, Georgia; 3 Department of Cardiac Surgery, Michigan Medicine, Ann Arbor, Michigan, USA

**Keywords:** Aortic dissection, COVID-19, Outcomes, Thoracic aortic repair

## Abstract

**Background:**

This study assesses impact of COVID-19 testing delay on perioperative outcomes of Acute Type A Aortic Dissection (ATAAD) repair at a single institution.

**Methods:**

From January 2010 – May 2021, 539 ATAAD patients underwent open aortic repair at our institution. Sixty-five of these patients had open aortic repair during COVID (March 2020 – May 2021) and 474 patients were pre-COVID (January 2010 – February 2020).

**Results:**

Compared to the pre-COVID group, patients During-COVID had a higher proportion of previous myocardial ischemia [9/65 (14%) vs 28/474 (5.9%), p=0.03], chronic obstructive pulmonary disease [14/65 (22%) vs 55/474 (12%), p=0.02], and renal malperfusion syndrome [11/65 (17%) vs 30/474 (6.4%), p=0.01]. There was no significant difference in surgical outcomes between groups, including operative mortality (7.6% vs 9.2%, p=0.64). The median admission-to-Operating Room (OR) time was 107 minutes in the During-COVID group compared to 87 minutes in pre-COVID group, p=0.88. During COVID, the median admission-to-OR time was significantly longer in the Waiting group compared to the No-waiting group (209 min vs 75min, p=0.0009). Only one patient had positive COVID test. There were no aortic ruptures while awaiting COVID testing results. There was a total of 6 reported deaths in the During-COVID group: 1 patient died post-surgery due to ARDS caused by COVID, and others due to ischemic stroke (3 patients) and organ failure (2 patients).

**Conclusions:**

Perioperative outcomes of ATAAD patients were similar during-COVID compared to pre-COVID. Waiting for COVID testing results did not significantly affect the perioperative outcomes among ATAAD patients after repair.

## 1. Introduction

SARS CoV-2 (COVID-19) virus is a highly infective pathogen associated with significantly increased operative mortality in cardiac surgery patients and high transmission risks to the medical team [1,2]. Guidelines to mitigate virus propagation has included COVID-19 virus testing for all patients prior to surgical procedures such as Acute Type A Aortic Dissection (ATAAD) repair [3–5]. However there is a dilemma if COVID-19 testing should take precedence over emergent ATAAD repair, and there is currently no evidence in literature to guide in this scenarios. Acute type A aortic dissection is a critical condition that requires swift surgical intervention due to high mortality rate of 1–2% per hour if left untreated after the onset of symptoms [6]. This study assesses impact of COVID-19 testing delay on perioperative outcomes in ATAAD patients after repair at a single institution.

## 2. Materials and Methods

This study was approved by the Institutional Review Board at Michigan Medicine (HUM001118517) and a waiver of informed consent was obtained.

### 2.1. Study Population

**Figure 1:**
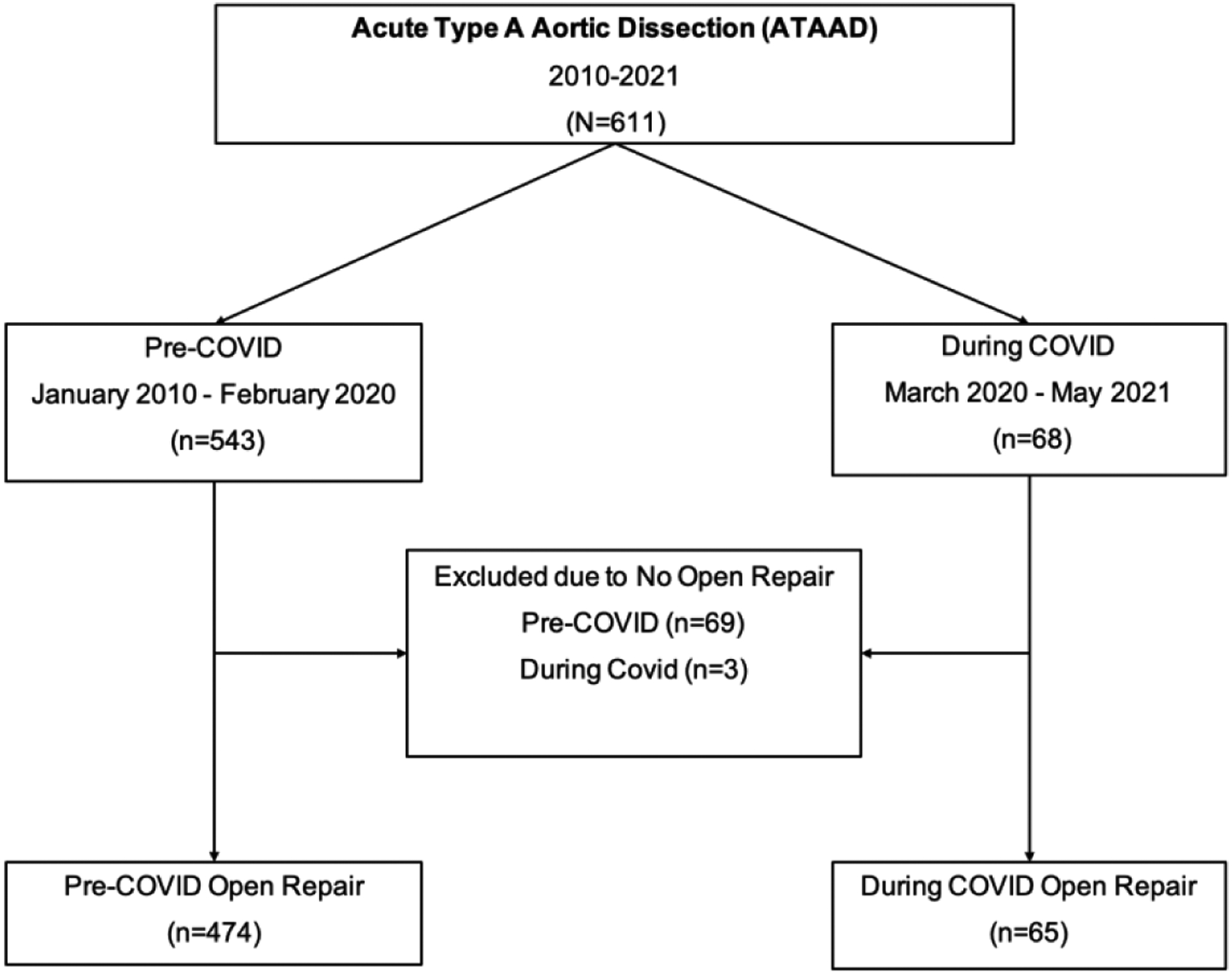
Consort diagram of selection and distribution of study population.

Between January 2010 – May 2021, 611 patients presented with acute type A aortic dissection at our institution. Sixty-eight patients during COVID (March 2020 – May 2021) and 543 patients pre-COVID (January 2010 – February 2020). In total only 539 patients had open ATAAD repair: During-COVID (n=65) and pre-COVID (n=474). All patients who did not undergo open aortic repair or poor surgical candidate were excluded from the study cohort: During-COVID group (n=3) and pre-COVID group (n=69), (Figure 1). All patients above 18years old who presented with ATAAD and underwent open surgical repair were included in the study. All patients during the COVID pandemic had COVID-19 testing using Reverse Transcriptase Polymerase Chain Reaction (RT-PCR). Waiting group included patients who waited for COVID-19 results prior to any open aortic repair or endovascular fenestration/stenting by interventional radiologist. Patients in the no-waiting group had COVID-19 testing done before transfer and the results of COVID test was known on admission. Data was retrieved through the University of Michigan Aortic Database to identify patients who underwent open surgical repair of ATAAD. Data from the Aortic Database was augmented with data from the Society of Thoracic Surgeons (STS) Michigan Medicine Cardiac Surgery Data Warehouse to determine preoperative, intraoperative, and postoperative characteristics. Univariate comparisons between groups were performed using Wilcoxon rank-sum tests for continuous data. The primary outcome was defined as perioperative outcomes in acute type A aortic dissection patients who waited for COVID-19 test results during the COVID-19 pandemic.

## 3. Statistical Analysis

Data were presented as median (25th, and 75th percentile) for continuous data and n (%) for categorical data. Comparisons of univariate characteristics were performed using Wilcoxon rank-sum tests for continuous data and chi-square tests for categorical data. Statistical calculations were performed using SAS 9.4 (SAS Institute, Cary, NC).

## 4. Results

### 4.1. Pre-COVID versus During-COVID group

#### 4.1.1. Preoperative demographics data

Compared to the pre-COVID group, patients During-COVID had a significantly higher proportion of chronic obstructive pulmonary disease (22% vs 12%), previous myocardial ischemia (14% vs 5.9%), peripheral vascular diseases (55% vs 28%), celiac malperfusion syndrome (9.2% vs 1.5%), renal malperfusion syndrome (17% vs 6.4%), lower extremity malperfusion syndrome (15% vs 7.5%), and DeBakey Class II (20% vs 10%) but lower proportion of patients with DeBakey Class I (80% vs 90%). Otherwise, there was no difference between groups (Table 1).

#### 4.1.2. Intraoperative and Perioperative Data

The median admission-to-operating room (OR/IR) time was similar between groups: 107 minutes in the During-COVID group compared to 87 minutes in pre-COVID group (p=0.88). The During-COVID group had higher proportion of aortic valve replacement (9.2% vs 2.4%), otherwise all other procedures such as aortic root replacement, root repair, arch replacement and concomitant mitral valve or tricuspid replacement were similar between groups. Compared to the pre-COVID group, patients During-COVID had shorter surgical incision-to-cardiopulmonary bypass (CPB) time (49minutes vs 76minutes), CPB time (163minutes vs 219 minutes), aortic cross clamp time (121minutes vs 144minutes) and hypothermic circulatory arrest (HCA) time (22minutes vs 31minutes), (Table 2). Lastly, compared to the pre-COVID group, the During-COVID group had higher proportion of deep sternal wound infection (4.7% vs 0.2%). Otherwise, there was no significant difference in surgical outcomes between groups, including operative mortality, acute renal failure requiring dialysis and stroke, among others (Table 3). Only one patient had positive COVID test. There was a total of 6 reported deaths in the During-COVID group: 1 patient died post-surgery due to ARDS caused by COVID, and others due to ischemic stroke (3 patients) and organ failure (2 patients).

### 4.2. Waiting versus no-waiting group during covid time

#### 4.2.1. Preoperative demographics data

Compared to the Waiting group, the No-waiting group had more patients with malperfusion syndrome (40% vs 10%) but patients with interventional radiology (IR) amenable malperfusion syndrome such as celiac, mesenteric, renal, and lower extremity malperfusion syndrome were similar between groups. Also, there was no difference in demographics between groups including chronic obstructive pulmonary disease, acute myocardial infarction, connective tissue disorder, cardiac tamponade, among others, (Table 4).

#### 4.2.2. Intraoperative and perioperative data

During COVID, the median admission-to-OR time was significantly longer in the Waiting group compared to the No-waiting group (209 minutes vs 75minutes). The average wait time for COVID-19 test result was 168 minutes. There were no aortic ruptures while awaiting COVID testing results. Furthermore, compared to the No-Waiting group, the Waiting group had less aortic root repair (50% vs 76%), but all other open aortic procedures including aortic root replacement, arch replacement, concomitant mitral and tricuspid valve replacement, and other intraoperative outcomes were similar between groups, (Table 5). Finally, postoperative outcomes such as operative mortality, pneumonia, prolonged ventilation, or acute renal failure requiring dialysis, were similar between the Waiting and No-Waiting group, (Table 6).

## 5. Discussion

It is well documented in literature that ATAAD is a catastrophic event and a surgical emergency with associated mortality rate of 1–2% per hour when untreated, but average operative mortality rate of 20–25% after repair [6,7]. Therefore, ATAAD patients historically undergo emergent surgical intervention to improve their survival outcome. However, the COVID-19 pandemic steered many hospitals and surgeons to require COVID-19 virus testing results prior to operative ATAAD repair [4,5]. One of the reasons for this decision is because COVID-19 virus has been associated with significantly increased operative mortality and morbidity in cardiac surgery patients, and knowledge of pre-operative infection status could help optimize patients to improve surgical outcomes [8,9]. Additionally, a multinational collaborative study (COVIDSurg Collaborative) reported a 34.0% 30-day postoperative mortality and 94.1% risk of pulmonary complication (pneumonia and acute respiratory distress syndrome) in COVID-19 positive cardiac surgery patients [2]. Furthermore, other rationales to wait for COVID-19 testing result is based on the evidence that preoperative patient testing limits healthcare workers exposure, minimize hospital acquired COVID-19 transmission to vulnerable patients and facilitate contact tracing in cases of exposure [10,11]. However, International Registry of Acute Aortic Dissection (IRAD) data showed that delayed ATAAD repair greater than 24 hours from symptom onset is associated with a 17.1% operative mortality in ATAAD patients [12]. Also, other studies have shown up to a 66.7% operative mortality in ATAAD patients delayed beyond 8–12 hours prior to repair [13]. Considering the risk associated with delayed surgical intervention in ATAAD patients as well as the risk and benefit of COVID-19 virus testing prior to ATAAD repair, our study sought to clarify the dilemma in the surgical management of acute Type A aortic dissection patients during COVID. In our study, all ATAAD patients had COVID-19 testing done at outside hospital before transfer or at our hospital before they went to the operating room except one patient in the early COVID pandemic. That patient eventually died of COVID respiratory failure after a straightforward open type A aortic dissection repair. Patients who waited for COVID-19 test had almost three times prolonged admission-to-operating room time compared to No-waiting group due to high COVID testing turnaround time (168 minutes). The prolong testing time could be because early technology and laboratory processes had not been streamlined. A major concern of delayed diagnosis and management of acute type A aortic dissection is the risk of aortic rupture [14]. However, in our study, there were no aortic ruptures while awaiting COVID testing results. This observation could be because of adequate medical stabilization such as blood pressure control prior to operative intervention. Also, we found that only one patient (0.02%) had a positive COVID-19 result prior to ATAAD repair. This patient did not wait for COVID result prior to intervention and died on postoperative day twelve due to Acute Respiratory Distress Syndrome (ARDS) and subsequent cardiac arrest. This case happened at early stage of pandemic. Risk of ARDS has been shown to be significantly increased in COVID-19 positive ATAAD patients after surgery due to the use of Cardiopulmonary Bypass (CPB). It is reported that CPB pump have non-endothelial surfaces that trigger proinflammatory responses mediated by tumor necrosis factor α and interleukin-10 [1,15]. This literature finding suggest that efforts could be directed towards perioperative management of inflammatory response in COVID-19 positive ATAAD patients to improve mortality outcomes associated with ARDS.

Finally, our study shows that operative mortality (i.e., both 30-day mortality and/or in-hospital mortality) was similar between ATAAD patients that waited for COVID-19 result prior to repair and No-waiting group. Our findings support that the operative delay in ATAAD patients due to prolonged COVID-19 testing did not worsen the surgical outcomes of ATAAD patients during COVID-19 pandemic at our institution. It is noteworthy that our results should be interpreted with caution due to small sample size in the waiting group and low study power, which could have resulted in our inability to delineate differences between groups. Also, our study is limited because it is a single center retrospective study and the timeline for the during-COVID group is short (March 2020-May 2021). We acknowledge that the COVID-19 pandemic is still ongoing and long-term studies would be warranted. In conclusion, our study showed that obtaining and waiting for COVID-19 test results during the COVID-19 pandemic delayed admission-to-operating room time in acute type A dissection patients but there was no change in perioperative outcomes. Institutional policy requiring mandatory COVID testing prior to acute type A dissection repair is appropriate and could protect vulnerable patients, limit healthcare workers exposure to symptomatic or asymptomatic COVID-19 carrier and facilitate contact tracing in cases of exposure.

## Figures and Tables

**Table 1:** Demographics and pre-existing conditions of Pre-COVID versus During-COVID group.

	Pre-COVID	During-COVID	p-value
	(n=474)	(n=65)	
Patient age (years)	60 (51, 69)	57 (48, 70)	0.65
Sex, male	313 (66)	44 (68)	0.79
BMI (kg/m2)	28 (25, 33)	27 (24, 31)	0.07
**Pre-existing comorbidities**			
Hypertension	367 (77)	57 (88)	0.06
Diabetes	39 (8.2)	10 (15)	0.06
Smoking status			0.41
Never	191 (41)	20 (31)	0.12
Former	157 (34)	27 (42)	0.2
Current	120 (26)	18 (28)	0.72
CAD	81 (18)	6 (9.2)	0.09
COPD	55 (12)	14 (22)	0.02
History of MI	28 (5.9)	9 (14)	0.03
History of renal failure	18 (3.8)	6 (9.2)	0.06
History of CVA	22 (79)	6 (9.2)	0.13
PVD	132 (28)	36 (55)	<0.0001
Connective tissue disorder	16 (3.4)	2 (3.1)	1.0
Previous cardiac surgery	37 (7.8)	6 (9.2)	0.69
**Aortic Insufficiency**			
Trace	49 (11)	8 (12)	0.71
Mild	125 (28)	14 (22)	0.31
Moderate	77 (17)	12 (18)	0.76
Severe	86 (19)	15 (23)	0.43

Ejection fraction	58 (55, 65)	60 (55, 65)	0.23
Acute MI	19 (4.0)	3 (4.6)	0.74
Acute stroke	42 (8.9)	7 (11)	0.63
Acute renal insufficiency	37 (7.8)	7 (11)	0.43
Acute paralysis	12 (2.6)	4 (6.2)	0.12
Cardiogenic shock	49 (10)	8 (12)	0.63
Tamponade	62 (14)	9 (14)	0.96
CPR	8 (1.7)	3 (5.3)	0.11
Preoperative creatinine	1.0 (0.8, 1.3)	1.1 (0.9, 1.4)	0.09
**Malperfusion syndrome**	116 (25)	20 (31)	0.29
Coronary	20 (4.3)	2 (3.1)	1
Cerebral	40 (8.5)	6 (9.2)	0.85
Spinal cord	7 (1.5)	2 (3.1)	0.3
Celiac	7 (1.5)	6 (9.2)	0.002
Mesenteric	34 (7.2)	7 (11)	0.32
Renal	30 (6.4)	11 (17)	0.01
Lower extremity	35 (7.5)	10 (15)	0.03

Delayed operation	48 (10)	9 (14)	0.37
DeBakey Class I	416 (90)	52 (80)	0.02
DeBakey Class II	47 (10)	13 (20)	0.02

Data presented as median (25%, 75%) for continuous data and n (%) for categorical data.

Abbreviations: AI=aortic insufficiency; BMI=body mass index; CAD=coronary artery disease; COPD=chronic obstructive pulmonary disease; CVA = cerebrovascular accident; MI=myocardial infarction, PVD = peripheral vascular disease. P-value indicates the difference between groups.

**Table 2:** Intraoperative Outcomes of Pre-COVID versus During-COVID group.

	Pre-COVID	During-COVID	p-value
	(n=474)	(n=65)	
Admission to OR/IR time (minutes)	87 (51, 251)	107 (42, 308)	0.88
Number of COVID tests per patient	0	1 (1,1)	
Waited for COVID test results	0	20 (31)	
**Aortic Root Procedure**			0.02
None	38 (8.1)	2 (3.1)	0.21
AVR only	11 (2.4)	6 (9.2)	0.01
Root replacement	143 (30)	13 (20)	0.11
Root repair	275 (59)	44 (68)	0.18
**Arch Replacement**			0.35
None	26 (5.5)	1 (1.5)	0.23
Hemiarch	288 (61)	46 (71)	0.14
Zone 1 Arch	41 (8.7)	5 (7.7)	0.78
Zone 2 Arch	88 (19)	8 (12)	0.2
Zone 3 Arch	26 (5.5)	5 (7.7)	0.57
Frozen Elephant Trunk	94 (20)	10 (15)	0.37
**Concomitant Procedures**			
CABG	24 (5.1)	1 (1.5)	0.34
Mitral valve	7 (1.5)	1 (1.5)	1
Tricuspid valve	6 (1.3)	1 (1.5)	0.6

Surgical incision to CPB (min)	76 (61, 96)	49 (35, 72)	0.02
CPB time (min)	219 (181, 271)	163 (126, 217)	<0.0001
Cross-clamp time (min)	144 (110, 198)	121 (82, 153)	0.0001
HCA	445 (95)	63 (98)	0.25
HCA time (min)	31 (23, 40)	22 (17, 33)	<0.0001
**Cerebral perfusion**			0.21
None	22 (4.7)	1 (1.6)	0.34
Antegrade	286 (61)	38 (59)	0.77
Retrograde	109 (23)	21 (33)	0.1
Both antegrade and retrograde	50 (11)	4 (6.3)	0.27

Lowest temperature (°C)	19 (18, 24)	25 (18, 27)	0.007
Blood transfusion (PRBCs), units	1.0 (0.0, 4.0)	0.0 (0.0, 2.5)	0.002

Data presented as median (25%, 75%) for continuous data and n (%) for categorical data.

Abbreviations: AVR=aortic valve replacement; CABG=coronary artery bypass graft; CPB=cardiopulmonary bypass; HCA=hypothermic circulatory arrest; PRBCs=packed red blood cells. P-value indicates the difference between groups.

**Table 3:** Postoperative Outcomes of Pre-COVID versus During-COVID group.

	Pre-COVID	During-COVID	p-value
	(n=474)	(n=65)	
Reoperation for bleeding	22 (4.7)	5 (7.8)	0.36
Tamponade	6 (1.3)	2 (3.1)	0.25
Deep sternal wound infection	1 (0.2)	3 (4.7)	0.006
Sepsis	13 (2.8)	4 (6.3)	0.14
Postoperative MI	3 (0.6)	0 (0)	1
Atrial fibrillation	156 (33)	24 (38)	0.52
Cerebrovascular accident	30 (6.4)	4 (6.3)	1
TIA	1 (0.2)	0 (0)	1
Acute renal insufficiency	59 (13)	12 (19)	0.18
Requiring dialysis	25 (5.4)	7 (11)	0.09
Permanent	12 (2.6)	2 (3.1)	0.68
Gastrointestinal complications	43 (9.2)	9 (14)	0.22
Pneumonia	69 (15)	14 (22)	0.14
Prolonged ventilation (>24 hours)	249 (53)	30 (47)	0.32
Hours intubated	28 (12, 74)	25 (10, 61)	0.3
Reintubation	31 (6.6)	6 (9.4)	0.43
Tracheostomy	10 (2.1)	2 (3.1)	0.65
Postoperative LOS (days)	10 (7, 16)	10 (7, 20)	0.68
Intraoperative mortality	3 (0.6)	0 (0)	1
In-hospital mortality	33 (7.0)	6 (9.2)	0.45
30-day mortality	36 (7.6)	6 (9.2)	0.62
Operative mortality*	36 (7.6)	6 (9.2)	0.64

Data presented as median (25 %, 75 %) for continuous data and n (%) for categorical data.

Abbreviations: LOS=length of stay; MI=myocardial infarction. P-value indicates the difference between groups.

* Operative mortality includes 30-day mortality and/or in-hospital mortality.

**Table 4:** Demographics and Preoperative Outcomes of During-COVID Group.

	No-Waiting	Waiting	p-value
	(n=45)	(n=20)	
Patient age (years)	56 (50, 69)	57 (48, 71)	0.84
Sex, male	28 (62)	16 (80)	0.15
BMI (kg/m2)	27 (23, 31)	28 (25, 31)	0.53
**Pre-existing comorbidities**			
Hypertension	37 (82)	0 (0)	0.05
Diabetes	5 (11)	5 (25)	0.26
Smoking status			0.49
Never	12 (27)	8 (40)	0.28
Former	14 (31)	4 (20)	0.36
Current	14 (29)	4 (25)	1
CAD	6 (13)	0 (0)	0.17
COPD	12 (27)	2 (10)	0.19
History of MI	8 (18)	1 (5.0)	0.25
History of renal failure	5 (11)	1 (5.0)	0.66
History of CVA	4 (8.9)	2 (10)	1
PVD	27 (60)	9 (45)	0.26
Connective tissue disorder	1 (2.2)	1 (5.0)	0.52
Previous cardiac surgery	5 (11)	1 (5.0)	0.66
**Pre-operative AI**			
Trace	3 (6.7)	5 (25)	0.09
Mild	11 (24)	3 (15)	0.52
Moderate	10 (22)	2 (10)	0.32
Severe	11 (24)	4 (20)	0.76

Ejection fraction	58 (55, 65)	65 (55, 67)	0.35
Acute MI	2 (4.4)	1 (5.0)	1
Acute stroke	4 (8.9)	3 (15)	0.67
Acute renal insufficiency	4 (8.9)	3 (15)	0.46
Acute paralysis	2 (4.4)	2 (10)	0.58
Cardiogenic shock	7 (16)	1 (5.0)	0.42
Tamponade	7 (16)	2 (10)	0.71
Preoperative creatinine	1.1 (0.9, 1.4)	1.2 (1.0, 1.3)	0.75
**Malperfusion syndrome**	18 (40)	2 (10)	0.02
Coronary	2 (4.4)	0 (0)	1
Cerebral	5 (11)	1 (5.0)	0.66
Spinal cord	2 (4.4)	0 (0)	1
Celiac	5 (11)	1 (5.0)	0.66
Mesenteric	5 (10)	2 (13)	1
Renal	8 (20)	2 (10)	0.48
Lower extremity	8 (18)	2 (10)	0.71
Delayed operation	7 (16)	2 (10)	0.71
DeBakey Class			
I	37 (82)	15 (75)	0.52
II	8 (18)	5 (25)	0.52

Data presented as median (25%, 75%) for continuous data and n (%) for categorical data.

Abbreviations: AI=aortic insufficiency; BMI=body mass index; CAD=coronary artery disease; COPD=chronic obstructive pulmonary disease; CVA = cerebrovascular accident; MI=myocardial infarction, PVD = peripheral vascular disease. P-value indicates the difference between groups.

**Table 5:** Intraoperative Outcome for During-COVID Group.

	No-Waiting	Waiting	p-value
	(n=45)	(n=20)	
Admission to OR/IR time (minutes)	75 (50, 176)	209 (187, 318)	0.0009
Number of COVID tests	1 (1,1)	1 (1,1)	1
Wait time for COVID test results (minutes)	0 (0,0)	168 (80, 186)	<0.0001
**Aortic Root Procedure**			0.02
None	0 (0)	2 (10)	0.09
AVR only	5 (11)	1 (5.0)	0.66
Root replacement	6 (13)	7 (35)	0.09
Root repair	34 (76)	10 (50)	0.04
**Arch Replacement**			0.9
None	1 (2.2)	0 (0)	1
Hemiarch	32 (71)	14 (70)	0.93
Zone 1 Arch	4 (8.9)	1 (5.0)	1
Zone 2 Arch	5 (11)	3 (15)	0.69
Zone 3 Arch	3 (6.7)	2 (10)	0.64
Frozen Elephant Trunk	8 (18)	2 (10)	0.71
**Concomitant Procedures**			
CABG	0 (0)	1 (5.0)	0.31
Mitral valve	1 (2.2)	0 (0)	1
Tricuspid valve	1 (2.2)	0 (0)	1

Surgical incision to CPB (min)	58 (47, 81)	31 (26, 35)	0.18
CPB time (min)	167 (126, 219)	149 (125, 204)	0.33
Cross-clamp time (min)	121 (82, 152)	115 (82, 175)	0.97
**HCA**			
HCA time (min)	24 (17, 33)	19 (17, 33)	0.44

**Cerebral perfusion**			0.14
None	1 (2.2)	0 (0)	1
Antegrade	29 (64)	9 (47)	0.2
Retrograde	14 (31)	7 (37)	0.66
Both antegrade and retrograde	1 (2.2)	3 (16)	0.07

Lowest temperature (°C)	24 (18, 27)	26 (22, 28)	0.09
Blood transfusion (PRBCs), units	0.0 (0.0, 3.0)	0.0 (0.0, 2.0)	0.21

Data presented as median (25%, 75%) for continuous data and n (%) for categorical data.

Abbreviations: AVR=aortic valve replacement; CABG=coronary artery bypass graft; CPB=cardiopulmonary bypass; HCA=hypothermic circulatory arrest; PRBCs=packed red blood cells. P-value indicates the difference between groups.

**Table 6:** Postoperative Data for During-COVID Group.

	No-Waiting	Waiting	p-value
	(n=45)	(n=20)	
Reoperation for bleeding	5 (11)	0 (0)	0.31
Tamponade	2 (4.4)	0 (0)	1
Deep sternal wound infection	2 (4.4)	1 (5.3)	1
Sepsis	2 (4.4)	2 (11)	0.58
Postoperative MI	0 (0)	0 (0)	1
Atrial fibrillation	16 (36)	8 (42)	0.62
Cerebrovascular accident	4 (8.9)	0 (0)	0.31
TIA	0 (0)	0 (0)	1
Acute renal insufficiency	10 (22)	2 (11)	0.48
Requiring dialysis	6 (13)	1 (5.3)	0.66
Permanent	2 (4.4)	0 (0)	1
Gastrointestinal complications	7 (16)	2 (11)	0.71
Pneumonia	11 (24)	3 (16)	0.53
Prolonged ventilation (>24 hrs)	24 (53)	6 (32)	0.11
Hours intubated	26 (10, 63)	16 (9, 61)	0.64
Reintubation	6 (13)	0 (0)	0.17
Tracheostomy	2 (4.4)	0 (0)	1
Postoperative LOS (days)	10 (7, 22)	9 (6, 17)	0.63
Intraoperative mortality	0 (0)	0 (0)	1
In-hospital mortality	6 (13)	0 (0)	0.17
30-day mortality	6 (13)	0 (0)	0.17
Operative mortality*	6 (13)	0 (0)	0.17

Data presented as median (25 %, 75 %) for continuous data and n (%) for categorical data.

Abbreviations: LOS=length of stay; MI=myocardial infarction. P-value indicates the difference between groups.

* Operative mortality includes 30-day mortality and/or in-hospital mortality.

## Abbreviations:

ATAAD: Acute type A aortic dissection
CPB: Cardiopulmonary bypass
SARS CoV-2 (COVID-19): Severe acute respiratory syndrome coronavirus 2
